# Genome Sequencing of Pathogenic *Rhodococcus* spp.

**DOI:** 10.3201/eid1811.120818

**Published:** 2012-11

**Authors:** Jun Hang, Robert J. Clifford, Yu Yang, Matthew C. Riley, Rupal M. Mody, Robert A. Kuschner, Emil P. Lesho

**Affiliations:** Walter Reed Army Institute of Research, Silver Spring, Maryland, USA (J. Hang, R.J. Clifford, Y. Yang, M.C. Riley, R.A. Kuschner, E.P. Lesho);; and William Beaumont Army Medical Center, El Paso, Texas, USA (R.M. Mody)

**Keywords:** Rhodococcus equi, Rhodococcus rhodochrous, Rhodococcus, rhodococci, bacteria, gram-positive, immunocompetent, lung, cavitary, genome sequencing, genome, sequence, DNA, RNA, United States

**To the Editor:** Infections caused by non-equi *Rhodococcus* spp. are uncommon but can cause severe pneumonia and bloodstream infections with sepsis (*1*–*4*). Increasing prevalence of immune-compromising illnesses and use of immunosuppressive agents might contribute to reemergence of these pathogens (*5*,*6*). Rhodococci infections may go undiagnosed or misclassified because of difficulties in laboratory identification, nomenclatural instability, and similarity of signs and symptoms to *Mycobacterium tuberculosis* infection (e.g., increasing cough, dyspnea, hemoptysis, and weight loss; failure to respond to broad-spectrum antimicrobial drugs; progressive cavitary lesion on repeated chest imaging).

A 73-year-old immunocompetent man with chronic obstructive pulmonary disease who lived on a ranch in the southwestern United States was evaluated for possible malignancy of the left lung. Computed tomography showed expansion of an upper lobe cavitary lesion, and he underwent a partial lobectomy. *Rhodococcus* spp. infection was suspected on the basis of the finding of the progressive lesion, salmon-pink colony growth on chocolate agar, and gram-positive coccobacilli on Gram stain. The isolate, R1101, could not be further identified by using commercial automated systems in the laboratory and so was subjected to genome study.

Alignment of 16S rRNA gene sequences showed that R1101 was most closely related to *R. rhodochrous*, with identities of 98.78%, 99.32%, and 99.86% to the 16S rRNA gene for type strains *R. rhodochrous* DSM43271, *R. pyridinivorans* PDB9, and *R. gordonae* W4937 (*7*), respectively. In contrast, sequence identity with *R. equi* type strain DSM20307 was 95.57%. Phylogenetic analysis with *Rhodococcus* 16S rRNA genes suggested that R1101 was a member of *R. rhodochrous* and evolutionarily separate from *R. equi* (Figure, panel A). A query against the National Center for Biotechnology Information nonredundant DNA database (http://blast.ncbi.nlm.nih.gov/Blast.cgi) found 60 rhodococcal 16S rRNA sequences that are 99%–100% identical to R1101, which demonstrates worldwide distribution of these closely related strains in diverse environments.

**Figure:**
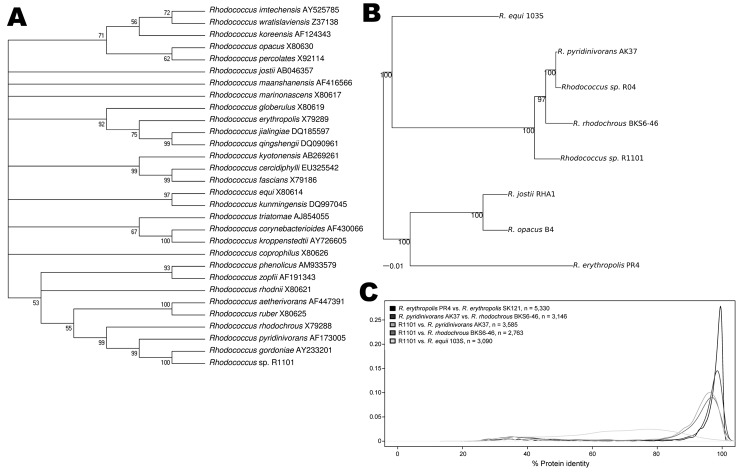
Phylogenetic and proteomic comparison of isolate R1101 and *Rhodococcus* spp. strains. A) 16S rRNA–based phylogenetic analysis. Complete or partial 16S rRNA gene sequences for 29 *Rhodococcus* spp. type strains were aligned with complete sequence of 16S rRNA gene for isolate R1101. GenBank accession numbers are provided. B) Genome-wide phylogenetic analysis. A sample of 67 protein-coding genes shared among genomes of isolate R1101 and 6 *Rhodococcus* spp. strains were analyzed. Scale bar indicates 0.01 nt substitutions per site. C) Genome-wide comparative proteome analysis. Density distributions of amino acid identity between homologous proteins from pairs of *Rhodococcus* strains are shown. The density distribution curves for R1101 show that R1101 is closely related, but not identical to, *R. pyridinivorans* (orange curve) and *R. rhodochrous* (red curve) at the protein level; R1101 is less closely related to *R. pyridinivorans AK37* and *R. rhodochrous* than these 2 species are related to each other; and R1101 is most distantly related to *R. equi* (green curve).

Optical genome mapping (*8*) of R1101 generated a consensus full-length map showing a circular chromosome map of ≈4.32 Mb (± 5%) in length. The whole-genome restriction map for R1101 was compared with in silico *Nco*I restriction maps of 33 complete genome sequences from the family *Nocardiaceae*, retrieved from GenBank; these sequences included *R. equi*. No substantial similarity (alignment scores >15%) was detected between R1101 and these genomes. Thus, we determined that R1101 was not an *R. equi* isolate.

Pyrosequencing of the R1101 genome yielded an average coverage depth of 14-fold. The total length of 1,020 assembled de novo contigs was 4.65 Mb, with a G + C content of 68%. A total of 3,969 putative protein-encoding genes were identified. Whole-genome phylogeny was used to show evolutionary distances between R1101 and the sequenced *Rhodococcus* species (Figure, panel B). We sampled ≈50.4 kb of sequences, encoding 67 putative proteins shared among all selected rhodococci, to generate the phylogenetic tree. Results confirmed that R1101 is a member of *R. rhodochrous* and is phylogenetically distant from *R. equi*.

We used a BLASTP search to determine the similarity of predicted R1101 proteins to homologous proteins from *R. equi* 103S, *R. erythropolis* (PR4 and SK121), and *R. pyridinivorans* (AK37 and BKS6-46) (Figure, panel C). These data indicate that R1101 is highly similar to but distinct from *R. pyridinivorans* and *R. rhodochrous* at the protein sequence level and is more distantly related to *R. equi*.

Each R1101 protein >1 database blast hit with an E-value <1 × 10^−10^ was assigned a best match to another species on the basis of its bit score. Consistent with the 16S rRNA–based phylogenetic analysis, >92% of the R1101 proteins showed greatest similarity to a protein from *R. pyridinivorans*, *R. rhodochrous*, or *Rhodococcus* sp. R04. However, 120 proteins (3%) have highest homology to their counterparts in the pathogen *R. equi*; 10 of these are unique to R1101 and *R. equi*. Furthermore, genes with the greatest similarity to *R. equi* are not randomly distributed throughout the R1101 genome.

We calculated the probability of observing groups of adjacent R1101 genes that are most similar to *R. equi* and observed 1 cluster of 5 adjacent genes (p = 2.33 × 10^−8^), 2 groups of 6 adjacent genes (p = 6.75 × 10^−10^), and 2 blocks of 7 contiguous genes (p = 1.94 × 10^−11^). Nucleotide BLAST analysis showed that R1101 sequence contigs 604, 456, 139, and 610 align to nt 4454241–4469589 of the *R. equi* 103S chromosome, with >97% identity at the DNA level. The 12 proteins wholly or partially encoded within this 15.3-kb region show >99% identity at the amino acid level with their R1101 orthologs. Among them, contig 139 encodes a cluster of 7 consecutive proteins with high homology to *R. equi*; this portion of the R1101 genome is likely to have been acquired from *R. equi* through horizontal gene transfer. R1101 may have acquired clusters of virulence-related genes, such as those crucial iron-uptake proteins (*9*), from the phylogenetically distant species *R. equi.*

In conclusion, infections caused by non-equi *Rhodococcus* spp. are rare, especially in immunocompetent patients, but may represent an emerging threat. Specialized diagnostics such as genome sequencing may be needed to accurately identify these pathogens.
